# Does Circadian Disruption Play a Role in the Metabolic–Hormonal Link to Delayed Lactogenesis II?

**DOI:** 10.3389/fnut.2015.00004

**Published:** 2015-02-23

**Authors:** Manjie Fu, Lingsong Zhang, Azza Ahmed, Karen Plaut, David M. Haas, Kinga Szucs, Theresa M. Casey

**Affiliations:** ^1^Department of Statistics, Purdue University, West Lafayette, IN, USA; ^2^School of Nursing, Purdue University, West Lafayette, IN, USA; ^3^Department of Animal Sciences, Purdue University, West Lafayette, IN, USA; ^4^Department of Obstetrics and Gynecology, School of Medicine, Indiana University, Indianapolis, IN, USA; ^5^Department of Pediatrics, School of Medicine, Indiana University, Indianapolis, IN, USA

**Keywords:** breastfeeding, chronodisruption, circadian clocks, delayed onset of lactogenesis II, lactation, metabolism, pregnancy, sleep

## Abstract

Breastfeeding improves maternal and child health. The American Academy of Pediatrics recommends exclusive breastfeeding for 6 months, with continued breastfeeding for at least 1 year. However, in the US, only 18.8% of infants are exclusively breastfed until 6 months of age. For mothers who initiate breastfeeding, the early post-partum period sets the stage for sustained breastfeeding. Mothers who experience breastfeeding problems in the early post-partum period are more likely to discontinue breastfeeding within 2 weeks. A major risk factor for shorter breastfeeding duration is delayed lactogenesis II (DLII; i.e., onset of milk “coming in” more than 72 h post-partum). Recent studies report a metabolic–hormonal link to DLII. This is not surprising because around the time of birth the mother’s entire metabolism changes to direct nutrients to mammary glands. Circadian and metabolic systems are closely linked, and our rodent studies suggest circadian clocks coordinate hormonal and metabolic changes to support lactation. Molecular and environmental disruption of the circadian system decreases a dam’s ability to initiate lactation and negatively impacts milk production. Circadian and metabolic systems evolved to be functional and adaptive when lifestyles and environmental exposures were quite different from modern times. We now have artificial lights, longer work days, and increases in shift work. Disruption in the circadian system due to shift work, jet-lag, sleep disorders, and other modern life style choices are associated with metabolic disorders, obesity, and impaired reproduction. We hypothesize that DLII is related to disruption of the mother’s circadian system. Here, we review literature that supports this hypothesis, and describe interventions that may help to increase breastfeeding success.

## Introduction

The World Health Organization recommends breast milk as the ideal food source for growth and development of infants (1). Human milk functions not only as food for the infant, but also protects against infection, promotes intestinal, immune, and cognitive development (2), and stimulates establishment of the unique gut microbiome (3, 4) of the breastfed infant. Breastfeeding also has beneficial effects on short- and long-term maternal and infant health outcomes. Teens and adults who were breastfed as babies are less likely to be overweight or obese and less likely to develop type-2 diabetes as well as perform better on intelligence tests (4). Mothers who breastfeed return to their pre-pregnancy weight faster, have lower rates of obesity, and lower risks of developing breast and ovarian cancers (1).

Due to the tremendous health benefits of breastfeeding, the American Academy of Pediatrics recommends exclusive breastfeeding (i.e., no supplementation with formula or solid food) for about 6 months, with continuation of breastfeeding for 1 year or longer as mutually desired by mother and infant (5). Economic analysis of breastfeeding benefits revealed that $13 billion in healthcare costs would be saved and 911 infant deaths prevented each year if 90% of families in the US complied with medical recommendations to breastfeed exclusively for 6 months (6). However, rates of adequate breastfeeding are far below national targets. The 2011 National Immunization Survey reported rates of breastfeeding initiation were at 79.2%, with breastfeeding rates dropping precipitously after that. Exclusive breastfeeding fell by 20%, to 59% at 1 week post-partum, 40.7% at 3 months, and only 18.8% of mothers exclusively breastfed for 6 months (7).

The most common reason mothers cite for stopping breastfeeding before their infant reached 2 weeks old, was that the baby was unsettled, a behavior often interpreted by mothers as indicating an insufficient milk supply (8). Delayed lactogenesis II (DLII), the onset of milk “coming in” more than 72 h post-partum, is a major contributor to early formula supplementation, inadequate breastfeeding, and breastfeeding cessation (9, 10). Further, infants of mothers who experience DLII are seven times more likely to lose excessive weight the first 5 days after birth (11).

## Lactogenesis in Women

Lactogenesis occurs in several stages. Lactogenesis I occurs during pregnancy and is the initiation of the synthetic capacity of the mammary glands. Lactogenesis II commences after delivery and is the initiation of plentiful milk secretion. Changes in milk composition from colostrum to mature milk in combination with a sudden feeling of breast fullness mark the onset of lactogenesis II, which normally occurs between 30 and 40 h following the birth of a full-term infant (10). Lactogenesis II is initiated post-partum by a fall in progesterone while prolactin levels remain high. The process does not depend on suckling of the infant until about the third or fourth day post-partum. Comparison between breastfeeding and non-breastfeeding women showed prolactin levels and milk secretion volumes are the same between groups of women the first 2 days post-partum (12, 13). Beginning day 3, post-partum prolactin levels begin to become significantly less in non-lactating women (12), and by day 4, secretion volume is lower in non-lactating women with lack of milk-removal initiating mammary involution and compositional differences in breast secretions between the groups (13). Thus, although breastfeeding is not necessary for initiation of lactogenesis II, it is essential for the continuation of lactation. The final stage of lactogenesis, lactogenesis III, also called galactopoiesis, is the production and maintenance of mature milk from day 9 post-partum, until weaning.

## Risk Factors for Delayed Onset of Lactogenesis II

Risk factors associated with DLII include primiparity, Cesarean delivery, longer duration of labor, and elevated blood cortisol concentrations (Table 1). The risk for low milk volume on day 4 post-partum was 4.3-fold (95% confidence interval-CI: 1.5–12.4) higher for mothers of pre-term infants delivered by Cesarean section versus vaginally (14). In this study, Cesarean delivery was associated with pregnancy-induced hypertension, delayed milk expression initiation, and low pumping frequency. Together, these findings suggest a composite of underlying risk factors contributes to the association of Cesarean delivery with DLII and low milk volume.

**Table 1:** **Risk factors for delayed or failed lactogenesis II [Modified from Ref. (10)]**.

**Delayed lactogenesis II**
Primiparity
Psychosocial stress/pain
Maternal obesity
Diabetes
Hypertension
Stressful labor and delivery
Cesarean section
Delayed first breastfeed episode
Low perinatal breastfeeding frequency
Elevated cortisol
**Failed lactogenesis II and/or low milk supply**
Breast surgery/injury
Retained placental fragments
Cigarette smoking
Hypothyroidism, hypopituitarism
Ovarian theca-lutein cyst
Insufficient mammary glandular tissue
Polycystic ovarian syndrome

Studies of primiparous women revealed that independent risk factors for DLII were maternal age ≥30 years, body mass index (BMI) in the overweight or obese range, and infant birth weight >3600 g (15). A dose-response relation to BMI was evident, with risk of DLII being 1.84 (95% CI: 1.02–2.80) times higher in overweight and 2.21 (95% CI: 1.52–4.30) times higher in obese women, as compared with women with a BMI in the healthy range (15). In obese women, DLII was not associated with psychosocial factors, such as planned duration of breastfeeding or behavioral beliefs about breast- and bottle-feeding (16). Therefore, it is likely that there is a physiological basis for the delay. Older maternal age and higher BMI are known risk factors for gestational diabetes (17). Lower glucose tolerance in the antenatal period was associated with longer time to onset of lactation (18), and prolactin release in response to suckling in the early post-partum period was found to be significantly lower in the overweight/obese women compared to healthy weight women (19). Importantly, low prolactin levels in women, as described for Sheehan’s syndrome, are associated with failed lactogenesis II (20). In addition, DLII often leads to failed lactogenesis II (14). Failed lactogenesis II is a condition wherein the mother is either able to achieve full lactation but an extrinsic factor has interfered with the process, or one or more factors results in failure to attain adequate milk production (10). Failed lactogenesis II can be described further in the context of two types of conditions: a primary inability to produce adequate milk volume, or a secondary condition as a result of improper breastfeeding management and/or infant-related problems (10).

## Metabolic-Hormonal Adaptations to Lactation

Lactation is the continuum of reproduction in mammals, and the most energetically demanding stage. Metabolically, the reproductive process in females can be divided into three periods which correspond to the energetic needs of the fetus and neonate. Period one spans the first two-thirds of pregnancy. There is little demand for nutrients by fetus during the first two trimesters, so the mother uses this time to store energy by increased consumption and enhanced lipogenesis (21). To support large gains in fetal growth, the mother transitions to a catabolic state in the last third of pregnancy, period two. Period two is characterized by increased gluconeogenesis, decreased peripheral tissue glucose utilization, increased fatty acid mobilization from adipose, and increased amino acid mobilization from muscle (22). Period three is lactation. During this period, the dam’s metabolism changes to accommodate the even greater energetic demands of milk synthesis. All the lactose and protein and most lipids in milk are synthesized in mammary gland, and thus the mammary gland has a high requirement for circulating substrates (glucose, amino acids, free fatty acids, and triglycerides) (21–23). In addition to further increasing metabolic responses described for period 2, there are substantial increases in size and complexity of the maternal intestine, liver, and cardiovascular system, including increased mammary blood flow, increased blood flow to liver and gastrointestinal tract, and higher cardiac output (24). Thus, the transition from pregnancy to lactation represents a major physiological change requiring on the one hand, coordinated changes in various body tissues, and on the other hand, mammary-specific changes to support a dominant physiological process (production of milk).

During pregnancy and at the onset of lactation, dramatic changes in circulating levels of reproductive and metabolic hormones (e.g., estrogen, progesterone, placental lactogen, prolactin, leptin, and cortisol) occur (12, 25). Hormonal changes stimulate metabolic changes in almost every organ of the body so that nutrients and energy can be diverted to the fetus to support growth before birth and then to the mammary gland to support milk synthesis post-partum (26, 27). Therefore, factors affecting metabolic-hormonal regulation (e.g., obesity, diabetes, hypothyroidism) during pregnancy, may also impact the ability of the mother to initiate lactation.

During pregnancy, the high levels of circulating progesterone enable differentiation of the mammary gland while inhibiting the secretory process of the mammary gland. Once the placenta is expelled after birth, progesterone levels decline rapidly, and increasing prolactin levels trigger the beginning of lactogenesis II (28). Neonatal suckling induces a neuroendocrine response that stimulates secretion of prolactin and glucocorticoids as well as oxytocin, which stimulates expulsion of milk from the gland (29). Increases in prolactin, estradiol, and cortisol levels during the periparturient period decrease peripheral tissue insulin sensitivity and responsiveness. These changes in insulin homeostasis result in increased rates of lipolysis and gluconeogenesis and decreased rates of glucose uptake by adipose and muscle, and decreased protein synthesis in muscle with concomitant increases in protein degradation and amino acid release (23, 30). Thyroid hormones are also essential for efficient milk production (31). A study of women with insufficient lactation found that the nasal administration of thyrotropin-releasing factor increased prolactin and daily milk volume (32).

## Hypothesis: Metabolic–Hormonal–Circadian Clock Link to Delayed Lactogenesis II

As outlined above, maternal hormonal milieu stimulates metabolic adaptations to reproductive state and mammary gland responsiveness. Therefore, it follows that conditions with a hormonal etiology (e.g., diabetes, hypothyroidism, or obesity) may interfere with these adaptations and cause a delay in lactogenesis II (10). Furthermore, some delivery modes and conditions that result in a delay in breastfeeding initiation and/or breast stimulation (e.g., pre-term, Cesarean, or a prolonged second stage of labor) may impact periparturient hormonal milieu needed to stimulate metabolic and mammary-specific adaptations needed to initiate copious milk secretion. We hypothesize that disruption of the circadian timing system during pregnancy and peripartum play a role in DLII.

The circadian timing system is intimately linked and reciprocally regulated by hormones and metabolism, and below we describe our preliminary studies that support this hypothesis. In addition, we summarize findings from a comprehensive database search in PubMed used to further support our hypothesis. In searching the literature to investigate this hypothesis, we found one of the immediate challenges encountered was the lack of studies conducted relating to the circadian timing system in pregnant or lactating women (33–36). In addition, information about what was considered normal or abnormal for circadian rhythms in pregnancy and lactation was lacking. Thus, much of the evidence used to develop and support our hypothesis was drawn from studies conducted on a more general population or inferred from animal studies.

## The Circadian Timing System

Nearly all physiological and behavioral functions of animals are rhythmic including secretion patterns of hormones, sleep–wake cycles, metabolism, and core body temperature. These circadian rhythms, 24 h cycles in biochemical, physiological, or behavioral processes, evolved as a common strategy among animals to coordinate internal systems and synchronize these systems to the environment (37, 38). Circadian rhythms are generated at the molecular level by circadian clocks. In mammals, circadian clocks are regulated hierarchically, with the master circadian clock located centrally in the suprachiasmatic nuclei (SCN) of the hypothalamus. In addition to the SCN, there are peripheral clocks distributed in every organ. The intrinsic rhythmicity of the SCN is entrained by synchronization to the 24-h day to regularly occurring environmental signals. The light–dark cycle is the most important environmental cue for entraining the master clock (39). Other cues include exercise, food availability, temperature, and stress, which directly or indirectly entrain the SCN (40, 41). The SCN integrates this temporal information and translates it into hormonal and autonomic signals that influence and synchronize peripheral clocks in every tissue of the body (42). In turn, peripheral clocks drive the circadian expression of local transcriptomes, thereby coordinating metabolism and physiology of the entire animal.

**Figure 1:**
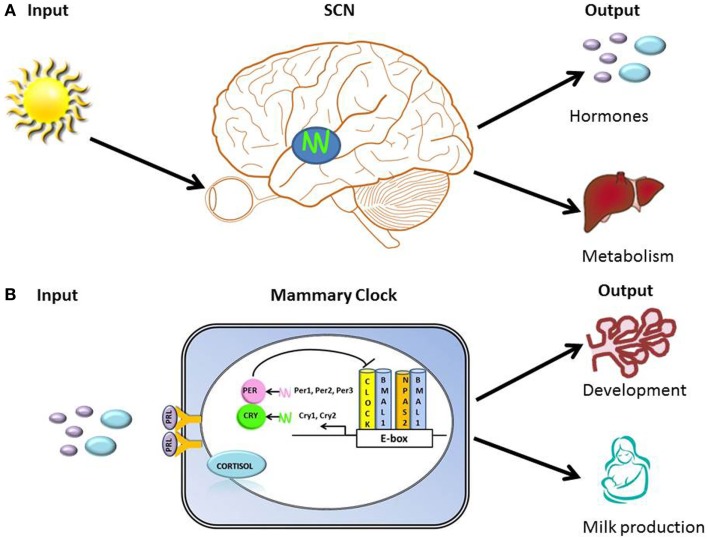
**Hallmarks of organization of clocks and circadian timing system**. **(A)** Illustration of primary input (light) to the SCN and outputs that include regulation of hormones (e.g., prolactin and cortisol) and metabolism. **(B)** Illustration of the transcription-translation feedback loop of the core molecular clock device, with proposed inputs [prolactin (PRL) and cortisol] and outputs (regulation of breast development and milk production) of mammary clock. At the core of the transcription-translation feedback loop are two transcription factors, CLOCK (or its ortholog NPAS2) and BMAL1, which in the form of a heterodimer drive rhythmic expression of output genes either directly via E-box regulatory element in their promoter regions, or indirectly by other transcription factors whose expression is under clock control (43). Among transcriptional targets of this complex are *Period* and *Cryptochrome* genes, whose products function as negative regulators of CLOCK/BMAL1-mediated transcription.

The circadian timing system must continuously adapt to and synchronize with the environment and the body’s internal signals in order to organize clocks into a coherent functional network that regulates behavior and physiology. Hallmarks of organization of circadian timing are the perception of environmental input, integration of time-related information into the circadian clock “device” (molecular clock), and transmission of adjusted timing information as output of metabolic and physiological processes (Figure 1). The molecular clock mechanism is based on a transcription-translation feedback loop. At the core of this loop are two transcription factors, CLOCK (or its ortholog NPAS2) and BMAL1, which in the form of a heterodimer drive rhythmic expression of output genes either directly via E-box regulatory element in their promoter regions, or indirectly by other transcription factors whose expression is under clock control (43). Among transcriptional targets of this complex are *Period* and *Cryptochrome* genes, whose products function as negative regulators of CLOCK/BMAL1-mediated transcription [Figure 1; (44)]. Approximately, 10–20% of genes expressed in a tissue exhibit circadian rhythms (45). Tissue-specific clock-controlled genes are involved in rate-limiting steps critical for organ function. For example, in the liver, molecules involved in metabolism of carbohydrate, lipid, and cholesterol encode genes that exhibit coordinated circadian expression (45). We propose that the mammary clock functions to regulate gland development and metabolic output [Figure 1; (46)].

Intimate interactions and reciprocal regulation occur between metabolic and circadian systems. The endogenous circadian timing system coordinates daily patterns of feeding, energy utilization, and energy storage across the daily 24 h cycle (47). Many metabolic hormones exhibit circadian rhythms. For example, cortisol levels are highest in the early morning and lowest at the first part of the biological night (47). Further, the SCN is responsible for a 24-h rhythm in plasma glucose concentrations, with the highest concentrations occurring toward the beginning of the activity period (48).

## Chronodisruption: Consequences to Metabolism and Health

Disruptions of normal circadian timing can evoke a multitude of downstream effects, including reorganizing the entire physiological state. Depressive mood (41), light, activity, and eating at night [e.g., night-shift work and night-eating syndrome; (49–53)], excessive weight (54), stress, and sleep disturbances (55) have all been characterized as chronodisruptors, i.e., factors that disrupt circadian rhythms. Circadian disruption can result in disorders such as diabetes, obesity, and cardiac disease (56–58). In humans, living in modern industrialized societies with 24 h access to light coupled with work and social obligations often leads to behaviors that are inappropriately timed relative to endogenous circadian rhythms. Night-shift work is an example of severe circadian disruption, as workers are awake, active, and eating during their biological night and trying to sleep and fast during their biological day (59).

Animal studies demonstrated that being active and feeding during the usual rest phase leads to alterations in metabolism and weight gain, even with the same caloric intake (60). In humans, internal desynchronization can be induced by a forced 28-h sleep–wake cycle (8 h sleep, 20 h awake), which is outside the range of entrainment for the human circadian clock (61). After four cycles, this protocol results in circadian misalignment, in which the behavioral sleep–wake cycle is 12 h out of phase with the circadian cycle. In these misaligned conditions, leptin rhythms are blunted, postprandial glucose and insulin are increased, and cortisol rhythms are 180° out of phase with the behavioral rhythm. Nearly half of the participants undergoing the 28-h cycle exhibited a pre-diabetic state during circadian misalignment (62).

Epidemiological studies have shown night-shift work, which disrupts the circadian system, is associated with development of obesity. Studies of women with phase-delayed eating patterns, such as not eating breakfast or night-eating syndrome, are associated with increased BMI, altered metabolism, changes in plasma hormone concentrations and rhythms, and depressive mood (52, 53). At the molecular level in humans, a single nucleotide polymorphism in CLOCK is associated with abnormal fatty acid metabolism and development of fatty liver, and a polymorphism in the BMAL1 core circadian clock gene is associated with susceptibility to hypertension and type-2 diabetes (63, 64).

In reciprocal, the over-fat state is characterized by alterations of circadian rhythms. In obese mice, there is attenuation of rhythmic gene expression patterns (65), and a delay in circadian entrainment to light-phase shift (66). Circadian rhythms of glucose and insulin are elevated in obese rats throughout the 24-h period. Levels of growth hormone, prolactin, and thyroxine are depressed. Serum levels of corticosterone do not exhibit distinct circadian rhythms and are elevated throughout the circadian cycle in obese rats (67). Similarly, in obese humans, basal levels of cortisol are higher with an attenuation of the circadian rhythm (68) and a lengthening of rhythm period (54).

With the advent of electric lighting, humans in industrialized societies are exposed to light at night. The natural light–dark cycle is the most salient cue for entraining the master clock to the 24-h day. The SCN communicates photoperiodic information to the pineal gland, where light inhibits melatonin secretion, such that melatonin secretion normally occurs at night. Melatonin has a fundamental role in regulating and timing several physiological functions, including glucose homeostasis, insulin secretion, and energy metabolism (69). As such, metabolism is impaired after a reduction in melatonin production, and the basic processes associated with acquisition and utilization of energy are functionally altered after exposure to extended periods of artificial lighting (70). Chronic light at night exposure suppresses melatonin levels as well as disrupts central clock rhythms, both of which are implicated in metabolic disturbances that predispose individuals to the development of type-2 diabetes, obesity, and metabolic syndrome (70). For example, a recent cross-sectional study of 500 people in Japan (71) found that elderly people sleeping in lighter rooms had higher body weight, waist circumference, and BMI; in that study, light exposure and obesity outcome variables were all objectively measured, although the BMI of participants was generally low (an average of 22.8). A large cohort study of 100,000 women revealed that the association between light at night exposure and obesity increased the odds of obesity with increasing levels of light at night exposure (72).

Sleep is cooperatively regulated by homeostatic and circadian factors. Voluntary sleep curtailment has become common in many modern life styles. For example, although the National Institutes of Health recommends that adults need 7–8 h of sleep per day (73), data from the National Health Interview Survey, found nearly 30% of adults reported an average of ≤6 h of sleep per day in 2005–2007 (74). Less than 1 week of sleep curtailment in healthy young men was associated with lower glucose tolerance, lower thyrotropin concentrations, and raised evening concentrations of cortisol (75). Poor sleep quality is also associated with increased risk for depression (76). Whereas short sleep duration is associated with increased incidence of diabetes, obesity (77), as well as increased all-cause mortality (78), there also appears to be a consistent association of poor sleep quality and short sleep duration with increased risk of cardiovascular disease, an association that is stronger in women than men (79, 80).

Disrupted sleep includes both abnormal sleep patterns and sleep deprivation. Studies have shown that disrupted sleep cycles impair the function of adipocytes, which regulate leptin levels (81). Abnormal leptin levels may lead to irregular meal times (82). This disrupts the balance between insulin and glucose cycles, causing reductions in insulin sensitivity and increases in glucose concentration, a prelude to diabetes (47, 81, 83). Lipid metabolism is similarly impaired, which may lead to increased lipogenesis, and by extension, obesity (82, 83). Impaired carbohydrate and lipid metabolism from disrupted circadian rhythms have also been linked to increased risk of cardiovascular disease (83, 84).

In human studies of shift work and atypical schedules, irregular sleep and disruptive circadian rhythms appear together, indicating that the two are closely related and that the presence of one usually entails the other (85, 86). In humans, sleep is normally timed to occur during the biological night, when body temperature is low and melatonin is synthesized. The sleep–wake cycle, and associated cycles of darkness and light and fasting and feeding, interacts with the circadian system and is a major driving factor of rhythms in physiology and behavior (87). Desynchrony of sleep-wake timing and other circadian rhythms, such as occurs in shift work and jet-lag, is associated with disruption of rhythmicity in physiology and endocrinology (87). Insufficient or mistimed sleep reduces the rhythmicity of clock-controlled transcripts and expression of core circadian clock genes. Thus, circadian disruption occurs as a result of irregular sleep patterns, (47, 82, 85, 86), and in converse circadian abnormalities can also result in sleep disturbances (88).

In summary, changes in glucose and lipid metabolism, abnormally high levels of cortisol at night, changes in melatonin, leptin, and thyroid hormone levels, as well as cardiovascular problems and development of type-2 diabetes are commonly associated with disruptions in circadian rhythms. Exposure to light, activity or eating at night, sleep disturbances/curtailment, depression, and stress are common chronodisruptors in many modern life styles and work schedules, and thus may be partly responsible for the rise in metabolic disease and obesity apparent in many industrialized societies (89).

## Circadian System Regulation of and Adaptations to Pregnancy and Lactation

As highlighted in multiple recent review articles (33, 90–92), much more work is needed to understand interactions among circadian clocks, metabolism, and female reproductive cycles and states. What is known, is that the circadian system plays a key role in the timing of reproductive events and hormones important to the regulation of pregnancy and lactation. For example, neural mechanisms regulating ovulation are under circadian control in many species to ensure that the timing of greatest fertility coincides with the period of maximal sexual motivation (93). SCN lesions result in infertility in rodents, due to the lack of the ability to synchronize events for ovulation (94), and mice with mutant core clock genes or core clock-gene knocked-out mice exhibit reduced fertility and fecundity (95, 96).

The SCN has been shown to be necessary for normal functioning of the hypothalamic-pituitary-gonadal (HPG) axis, and rhythms of clock-gene expression have been recorded in brain regions controlling both the HPG and hypothalamic-pituitary-adrenal (HPA) axis (97). Rhythmic gene expression of prolactin in pituitary mammotrophs was shown to be mediated by CLOCK–BMAL1 binding to clock-gene regulatory elements (98). In addition, ovariectomized and estradiol-treated rats fail to exhibit a prolactin surge following SCN lesions. Furthermore, SCN lesion also prevents the twice daily prolactin surge induced by mating in rodents, which maintains the corpus luteum and thus the secretion of progesterone and pregnancy maintenance [for review, see Ref. (93)], suggesting the central clock plays a direct or indirect role in regulation of prolactin secretion. Therefore, it is interesting to speculate that decreased blood prolactin observed in healthy, young non-pregnant women following exposure to partial sleep deprivation (99) is due to the disruption of the master clock.

Circadian rhythms in behavior and physiology change substantially as female mammals transition through the reproductive states of non-pregnancy, pregnancy, and lactation, with changes in circadian rhythms supporting physiological demands unique to each of these stages (100–104). For example, to compensate for increases in the daily temperature minimum during gestation studies of pregnant laboratory animals showed phase of body temperature rhythm was advanced and amplitude decreased relative to non-pregnant controls (100). Further, to compensate for the increased need for sleep in early pregnancy, sleep patterns are altered in pregnant rodents (103, 105).

Sleep is also significantly impacted by pregnancy in women. A study of 192 pregnant women surveyed retrospectively found 88% had alterations in sleep compared with their usual experience (106). Reported changes included insomnia, parasomnias (nightmares and night terrors), restless leg syndrome, snoring, and sleep apnea. Among the most frequent self-reported causes of sleep disturbance during pregnancy were urinary frequency, back or hip ache, and heartburn. A prospective, cohort study of healthy nulliparous women found compared with the baseline assessment done before 20 weeks gestation, mean sleep duration in the third trimester was significantly shorter (7.4 h compared with 7.0 h), and overall poor sleep quality became significantly more common as pregnancy progressed (107). Okun and Coussons-Read collected qualitative sleep data at 12, 24, and 36 weeks’ gestation, and found as early as 12 weeks, pregnant women reported an increased number of naps, nocturnal awakenings, time spent awake during the night, and poorer sleep quality than non-pregnant women (108).

The dramatic fluctuations in reproductive hormones that occur during pregnancy and the transition from pregnancy to the post-partum period are accompanied by alterations in circadian rhythms of melatonin (109, 110) and cortisol (111). In seasonally breeding animals, melatonin regulates reproductive hormones and behavior (112). During pregnancy in humans, night time melatonin levels increase linearly with progressive weeks of gestation, and fall in the early post-partum period (110). Changes in cortisol dynamics during pregnancy are due in part to the remodeling of maternal HPA axis, which results in an altered maternal stress response and energy balance, as well as rising placental cortico-releasing hormone (CRH) levels (113). Change in the HPA axis and placental CRH result in attenuated rhythms of plasma cortisol and a period of hypercortisolism beginning in mid-gestation. Following birth of the neonate, maternal plasma levels of cortisol drop due to loss of placental CRH, if the mother breastfeeds, attenuation in cortisol rhythms and stress response continue throughout lactation (114, 115). Synchronization among the multitude of molecular clocks in the body is believed to be regulated in part by cortisol circadian rhythms which are regulated by the central clock (116). Thus, changes in cortisol secretion patterns during pregnancy and lactation have the potential to affect circadian rhythms across the entire body.

Timing of parturition in women also shows signs of being regulated by the circadian timing system. For example, the onset of labor and spontaneous membrane rupture peaks at night between midnight and 4:00 a.m. (117–119), and the timing of births peak around 1:00–2:00 p.m. for primiparous women (120). Further, a nested, randomized, controlled clinical trial that compared morning versus evening administration of prostaglandin and its success rate in inducing labor, reported no difference in rate of Cesarean delivery, however morning inductions required less oxytocin, had a shorter induction to birth interval, and were less likely to result in instrumental vaginal births for primiparous mothers (121).

During lactation in women, the potent lactogens, prolactin and cortisol, exhibit circadian variation in secretion. The prolactin-secretory response to nursing is superimposed on the endogenous circadian rhythm of prolactin secretion, thus the suckling stimulus elevates prolactin levels more effectively at certain times of day when the circadian input enhances the suckling stimulus-evoked secretory response (122). Studies in lactating rabbits revealed timing the single bout of daily suckling that occurs in this species shifted PER1 expression in SCN clock and in peripheral clocks of the brain (76, 77). Our *in vitro* studies showed prolactin and glucocorticoids can directly affect mammary clock, with prolactin inducing phase shifts in core clock genes expression, suggesting that external cues emanating from neonate can have effects on maternal circadian physiology.

Our rodent studies also demonstrated that during the transition from pregnancy to lactation, dynamic changes in core clocks occurred in multiple tissues. The amplitude of core clock genes’ expression increased significantly in the SCN and liver (123). Work of others found that expression of PER2 expression shifted and amplitude increased in SCN in early pregnant versus diestrus rats (124). The central clock functions to synchronize the timing of metabolic and reproductive functions, and thus changes in the SCN during the transition in physiological states may function to mediate coordinated changes in tissue-specific metabolism needed to support pregnancy and lactation. Increases in amplitude of hepatic expression of core clock genes’ rhythms during the transition from pregnancy to lactation, likely reflect the increase in liver metabolic output (123). In addition, changes revealed in mammary clock dynamics led us to hypothesize that differentiation-driven changes during the transition from pregnancy to lactation in the mammary clock are stimulated, in part, by peripartum changes in prolactin and glucocorticoids. Further, we envision that differentiation-associated changes in mammary clock mediate the increase in metabolic output of the gland during lactation (123).

Milk synthesis and composition shows circadian variation in lactating women (24). Approximately, seven percent of the genes expressed in the lactating breast show circadian oscillation; many of these genes regulate cell growth and differentiation as well as metabolic pathways (125). Offspring of homozygous female *Clock-Δ19* mutant mice fail to thrive suggesting that the mutation affects the dam’s ability to support milk production during lactation. Our studies of *ClockΔ19 mice* revealed poorer mammary development and evidence for delayed or failed lactogenesis II, with *in vitro* studies demonstrating a role for *Clock* in regulating mammary epithelial growth and differentiation (unpublished data). Miller et al. have evidence to suggest that prolactin release is altered in *ClockΔ19 mice* (126). Thus, both systemic and mammary-specific alterations likely account for negative impact of *ClockΔ19* mutation on lactation. The photoperiod effect on ruminant milk production (127) and our studies with cattle showing circadian disruption significantly decreases milk production (46), also support a role for the circadian timing system in mediating systemic metabolism and mammary metabolic output during lactation.

## Consequences of Chronodisruption on Ability of Mother to Support Offspring

Several rodent studies have been designed to determine if circadian disruption impacts pregnancy outcome. These studies found that exposing mice immediately after confirmed mating to continuous shifts in the light-dark cycle (a chronic jet-lag model) resulted in a significant decrease in the number of full-term pregnancies (128). Rat dams exposed to chronic jet-lag throughout gestation gained 70% less weight during the first week of pregnancy than those housed in control conditions. In late pregnancy (gestation day 20), chronic jet-lag exposure profoundly disrupted timing of corticosterone, leptin, glucose, insulin, free fatty acids, triglycerides, and cholesterol concentrations in these dams. Further, expression of gluconeogenic and circadian clock genes in maternal and fetal liver was arrhythmic relative to controls (129). Offspring of rat dams exposed to a chronic jet-lag paradigm from the first day of pregnancy to lactation day 10 developed metabolic problems such as obesity, hyperleptinemia, and glucose tolerance/insulin insensitivity when they reached maturity (130). These studies demonstrate that exposure to chronic circadian disruption during pregnancy impacts the normal maternal metabolic-hormonal adaptations to this physiological state. Further, these perturbations may contribute to the programing of poor metabolic homeostasis in adult offspring.

In humans, a polymorphism in the circadian clock-gene BMAL1 was shown to be associated with increased risk of miscarriages (131). Studies of shift workers found night and rotating shift work during pregnancy increased the risk of pre-term birth, low birth weight, and miscarriage (132, 133). For example, a retrospective study of a large cohort in women (National Birth Cohort in Denmark) reported a fixed night work schedule increases risk of post-term birth (odds ratio, 1.35; 95% CI, 1.01–1.79). Fixed evening work had a higher risk of full-term low birth weight (odds ratio, 1.80; 95% CI, 1.10–2.94); and shift work as a group showed a slight excess of small-for-gestational-age babies (odds ratio, 1.09; 95% CI, 1.00–1.18) (134). A population-based prospective cohort study conducted in Sri Lanka found risk factors for small-for-gestational-age were shift work and exposure to physical and chemical hazards during second and third trimesters (odds ratio, 4.20; 95% CI, 1.10–16.0), as well as sleeping ≤8 h during second or third trimesters (odds ratio, 2.23; 95% CI, 1.08–4.59) (135).

A prospective cohort study of approximately 1,200 healthy pregnant women was used to evaluate the influence of maternal self-reported sleep duration during early pregnancy on blood pressure levels and risk of hypertensive disorders of pregnancy. Investigators found that the mean third trimester systolic blood pressure was higher for women reporting ≤6 and 7–8 h sleep compared with women reporting 9 h of sleep, with odds ratio for pre-eclampsia in very short (<5 h) sleepers being 9.52 (95% CI, 1.83–49.40) (136). Sleep disturbances in early pregnancy are also associated with higher risk for development of hyperglycemia (137). Moreover, gestational diabetes mellitus risk was increased among women sleeping <4 h compared with those sleeping 9 h per night during early pregnancy with relative risk for overweight women threefold higher (138). Snoring, which is associated with sleep disturbances, was associated with a 1.86-fold (95% CI, 0.88–3.94) increased risk of gestational diabetes, with the risk being 6.9-fold (95% CI, 2.87–16.6) higher in overweight women who snored compared with lean women (138). Hyperleptinemia is also an important clinical risk factor for adverse pregnancy outcomes such as pre-eclampsia and gestational diabetes mellitus (139–141). A cross-sectional study of 830 pregnant women found that shorter sleep (≤5 h) and longer sleep (≥9 h) were associated with elevated leptin among overweight or obese women (142).

**Figure 2:**
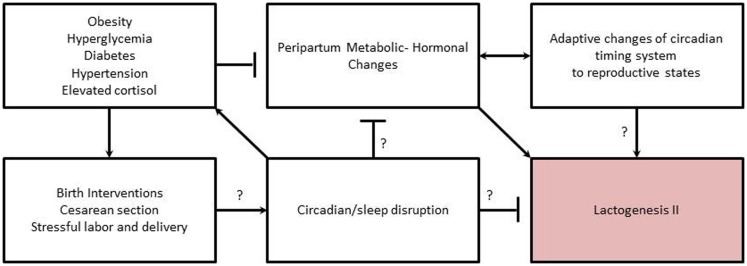
**Proposed relationships among metabolic-hormonal-circadian clock changes needed to initiate lactogenesis II during the periparturient period, and how related risk factors interfere with initiation**. Arrows indicate inductive relationship. “T” indicates an inhibitory relationship. Question marks indicate areas needing further research to support proposed relationship/mechanism.

Researchers have also linked abnormalities in circadian rhythms with development of mood disorders such as bipolar disorder, major depression, and seasonal affective disorder (143, 144). Individuals with major depression exhibit blunted or abnormal circadian rhythms in body temperature, plasma cortisol, norepinephrine, thyroid stimulating hormone, blood pressure, pulse, and melatonin (143). Studies of depressed pregnant women found significantly lower levels and phased-advanced melatonin secretion in pregnant women with personal and family histories of depression relative to women without history of depression (110). Further, in healthy women, plasma melatonin levels became increasingly elevated as pregnancy progressed but this increase did not occur in depressed women (110). Thus, it is interesting to speculate whether mothers with depression are at an increased risk for shorter breastfeeding duration and increased breastfeeding difficulties (145), in part, through physiological disruption of the circadian timing system, which in turn impacts her milk production (Figure 2).

The association of maternal obesity with DLII appears to have a physiological basis related to alterations in hormones and metabolic adaptations needed to initiate copious milk production (Figure 2). In rodent models of obesity, the normal hormonal response to the periparturient period is altered, with a lower rise in prolactin and insulin levels during the transition from pregnancy to lactation and significantly higher corticosterone levels (146–148). In obese humans, basal levels of cortisol are also higher with an attenuation of the circadian rhythm (68) and a lengthening of rhythm period (54). Circadian rhythms of plasma cortisol are believed to be a primary signal for synchronization of peripheral clocks (149). Glucocorticoids also regulate milk synthesis (150). However, antenatal treatment with glucocorticoids delays secretory activation in ewes (151), and treatment of animals with supra-physiological of glucocorticoids depresses milk production in an established lactation (150). Thus, the delay in onset of lactogenesis II experienced by obese women may be due to alterations in coordinated changes and interactions among circadian timing system, endocrine milieu, and metabolism needed to initiate copious milk secretion (Figure 2).

## Conclusion and Potential Interventions

Although there is a paucity of information available to understand the role of the circadian timing system in mediating metabolic and hormonal adaptations to pregnancy and lactation, there is strong evidence that clocks play a role in regulating metabolic and hormonal homeostasis in animals. The circadian system functions to prepare physiological systems and behavioral activity for anticipated changes in the environment (e.g., day–night cycle). In addition, the circadian system also prepares for anticipated changes in physiological–reproductive state (e.g., seasonal fertility in some species). Chronic disruption of the circadian timing system has negative impacts on fertility and fecundity in females. Fertility and fecundity depend on precise hormonal timing and adequate metabolic adaptions to support the extra energy investment of reproduction (92, 152). Similarly, the initiation of lactogenesis II, requires timing coordinated changes in hormones and metabolism to initiate copious milk production in the early post-partum. Thus, we hypothesize that chronic disruption of the maternal circadian timing system during pregnancy and peripartum alters hormones and metabolic adaptations resulting in DLII (Figure 2).

Human lactation is a complex phenomenon and the initiation and duration of breastfeeding is influenced by many demographic, physical, social, and psychological variables. Interventions developed to increase the rates of successful breastfeeding target management strategies to ensure adequate milk supply (153–155). Although there is evidence to suggest that the circadian system plays a significant role in lactation and maternal behavior, current breastfeeding interventions do not encompass management strategies and education that take into account circadian disruptions. Depressive mood, light, activity, and eating at night (e.g., night-shift work and night-eating syndrome), excessive weight, and sleep disturbances are well characterized chronodisruptors. These chronodisruptors have also been associated with hormonal and metabolic alterations during pregnancy and inadequate breastfeeding outcomes.

Therefore, we propose the need to test interventions aimed at maintaining circadian alignment (e.g., limiting exposure to chronodisruption) during three stages that impact the ability of the mother to initiate and maintain lactogenesis: (1) during pregnancy; (2) in the hospital; and (3) after post-partum discharge from the hospital. Interventions during pregnancy may include, raising mothers’ awareness of their sleep, eating and exercise patterns through diaries and self-monitoring, as well and educating mothers about sleep hygiene and consequences of sleep deprivation and interrupted sleep cycle and exposure to light at night.

Good sleep hygiene, together with circadian alignment of food intake, a regular meal frequency, as well as attention for protein intake or diets, may contribute to cure sleep abnormalities and overweight/obesity (47). Circadian alignment diminishes the urge to overeat, normalizes substrate oxidation, stress, and insulin and glucose metabolism. In addition, circadian alignment impacts leptin concentrations, lipid metabolism, blood pressure, appetite, energy expenditure, and substrate oxidation, and normalizes the experience of food reward (47). For example, a clinical trial investigated whether sleep extension under real-life conditions is a feasible intervention in 16 healthy non-obese adults who were chronically sleep restricted (156). The intervention was 2 weeks of habitual time in bed, followed by 6 weeks during which participants were instructed to increase their time in bed by 1 h per day. Continuous actigraphy monitoring and daily sleep logs during the entire study showed that sleep time during weekdays increased (mean actigraphic data: 44 ± 34 min, *P* < 0.0001; polysomnographic data: 49 ± 68 min, *P* = 0.014), without any significant change during weekends. Changes from habitual time in bed to the end of the intervention in total sleep time correlated with changes in glucose and insulin levels, as well as with indices in insulin sensitivity.

In the hospital, interventions may include implementing light–dark cycles and/or light filters that help to maintain circadian alignment; educating families about the importance of limited visiting hours and number of visitors; and implementing Baby Friendly Hospital Initiative (157) to include quiet time and light-dark cycles. Discharge interventions may include providing education about sleep hygiene, diet, and activities important to maintain circadian alignment.

Exposure to lighting environments that more closely align to the Earth’s natural light–dark cycles may prove to limit metabolic-hormonal disturbances during pregnancy and promote normal metabolic-hormonal adaptations during the peripartum needed to initiate lactation. An example of the recognition that hospital lighting environment impacts physiology, health, and development, comes from studies of infants in Neonatal Intensive Care Units (NICU) [for review, see Ref. (158)]. Providing a light–dark cycle in the NICU increased sleeping time in infants, decreased the time spent feeding, and increased weight gain resulting in earlier hospital discharge relative to infants exposed to constant lighting typical of some hospital nurseries (159, 160). In addition, exposure to a light–dark cycle promoted heart rate stability, improved oxygen saturation, establishment of daily melatonin rhythms, and a better tolerance to milk (160). These studies demonstrate that exposure to a light–dark cycle immediately after birth promotes beneficial effects on the development of infants, and thus support the need for research on the impact of hospital lighting environment on maternal physiology and maternal-offspring interactions in the peripartum that affect breastfeeding outcomes.

Recent studies using animal and clinical models have demonstrated that filtering short wavelengths (below 480 nm) for nocturnal lighting can attenuate alterations in hormone secretion (melatonin and glucocorticoids), and in central and peripheral clock-gene expression induced by nighttime light exposure (161). In humans, use of optical filters led to an improvement in mood and cognitive performance under controlled laboratory conditions as well as during field-based shiftwork studies. For example, studies found that use of optical filters during shift work increased sleep duration and quality on nights immediately following night shifts (161). Thus, a method to improve or prevent many of the health problems associated with circadian misalignment, including timing to onset of lactogenesis II may be to incorporate optical filters into glasses or as coverings for light bulbs in work places and hospitals for procedures that require night time exposure to light (161).

If these proposed interventions prove to mitigate the development of metabolic and hormonal imbalances that increase the risk of DLII, the rates of adequate breastfeeding may increase. Importantly, since many of the external factors that disrupt circadian clocks are modifiable by changes in lifestyle or external environment, the interventions we suggest are minimally invasive and thus are readily implementable during pregnancy and peripartum.

## Conflict of Interest Statement

The authors declare that the research was conducted in the absence of any commercial or financial relationships that could be construed as a potential conflict of interest.

## References

[1] Ten Facts about Breastfeeding. World Health Organization (2014). Available from: http://www.who.int/features/factfiles/breastfeeding/en/

[2] Lassek WD Gaulin SJC. Maternal milk DHA content predicts cognitive performance in a sample of 28 nations. Matern Child Nutr (2013). 10.1111/mcn.12060 23795772 PMC6860246

[3] Zivkovic AM German JB Lebrilla CB Mills DA. Human milk glycobiome and its impact on the infant gastrointestinal microbiota. Proc Natl Acad Sci U S A (2011) 108(Suppl 1):4653–8. 10.1073/pnas.1000083107 20679197 PMC3063602

[4] Neville M Anderson S McManaman J Badger T Bunik M Contractor N Lactation and neonatal nutrition: defining and refining the critical questions. J Mammary Gland Biol Neoplasia (2012) 17:167–88. 10.1007/s10911-012-9261-5 22752723 PMC3428522

[5] Johnston M Landers S Noble L Szucs K Viehmann L. Breastfeeding and the use of human milk. Pediatrics (2012) 129:e827–41. 10.1542/peds.2011-3552 22371471

[6] Bartick M Reinhold A. The burden of suboptimal breastfeeding in the United States: a pediatric cost analysis. Pediatrics (2010) 125:e1048–56. 10.1542/peds.2009-1616 20368314

[7] Breastfeeding Among U.S. Children Born 2001–2011, CDC National Immunization Survey. (2014). Available from: http://www.cdc.gov/breastfeeding/data/NIS_data/index.htm

[8] Colin WB Scott JA. Breastfeeding: reasons for starting, reasons for stopping and problems along the way. Breastfeed Rev (2002) 10:13–9. 12227559

[9] Brownell E Howard CR Lawrence RA Dozier AM. Delayed onset lactogenesis II predicts the cessation of any or exclusive breastfeeding. J Pediatr (2012) 161:608–14. 10.1016/j.jpeds.2012.03.035 22575242 PMC3670592

[10] Hurst NM. Recognizing and treating delayed or failed lactogenesis II. J Midwifery Womens Health (2007) 52:588–94. 10.1016/j.jmwh.2007.05.005 17983996

[11] Dewey K Nommsen-Rivers L Heinig M Cohen R. Risk factors for suboptimal infant breastfeeding behavior, delayed onset of lactation, and excess neonatal weight loss. Pediatrics (2003) 112:607–19. 10.1542/peds.112.3.607 12949292

[12] Neville MC Morton J. Physiology and endocrine changes underlying human lactogenesis II. J Nutr (2001) 131:3005S–8S. 11694636 10.1093/jn/131.11.3005S

[13] Kulski JK Hartmann PE. Changes in human milk composition during the initiation of lactation. Aust J Exp Biol Med Sci (1981) 59:101–14. 10.1038/icb.1981.66 7236122

[14] Murase M Nommsen-Rivers L Morrow AL Hatsuno M Mizuno K Taki M Predictors of low milk volume among mothers who delivered preterm. J Hum Lact (2014) 30:425–35. 10.1177/0890334414543951 25063573

[15] Nommsen-Rivers LA Chantry CJ Peerson JM Cohen RJ Dewey KG. Delayed onset of lactogenesis among first-time mothers is related to maternal obesity and factors associated with ineffective breastfeeding. Am J Clin Nutr (2010) 92:574–84. 10.3945/ajcn.2010.29192 20573792

[16] Hilson JA Rasmussen KM Kjolhede CL. High prepregnant body mass index is associated with poor lactation outcomes among white, rural women independent of psychosocial and demographic correlates. J Hum Lact (2004) 20:18–29. 10.1177/0890334403261345 14974697

[17] Aviram A Hod M Yogev Y. Maternal obesity: implications for pregnancy outcome and long-term risks–a link to maternal nutrition. Int J Gynecol Obstet (2011) 115(Suppl 1):S6–10. 10.1016/S0020-7292(11)60004-0 22099446

[18] Nommsen-Rivers L Dolan L Huang B. Timing of stage II lactogenesis is predicted by antenatal metabolic health in a cohort of primiparas. Breastfeed Med (2012) 7:43–9. 10.1089/bfm.2011.0007 21524193 PMC3546359

[19] Rasmussen KM Kjolhede CL. Prepregnant overweight and obesity diminish the prolactin response to suckling in the first week postpartum. Pediatrics (2004) 113:e465–71. 10.1542/peds.113.5.e465 15121990

[20] Ramiandrasoa C Castinetti F Raingeard I Fenichel P Chabre O Brue T Delayed diagnosis of Sheehan’s syndrome in a developed country: a retrospective cohort study. Eur J Endocrinol (2013) 169:431–8. 10.1530/EJE-13-0279 23864341

[21] Herrera E. Metabolic adaptations in pregnancy and their implications for the availability of substrates to the fetus. Eur J Clin Nutr (2000) 54(Suppl 1):S47–51. 10.1038/sj.ejcn.1600984 10805038

[22] Bauman DE Currie WB. Partitioning of nutrients during pregnancy and lactation: a review of mechanisms involving homeostasis and homeorhesis. J Dairy Sci (1980) 63:1514–29. 10.3168/jds.S0022-0302(80)83111-0 7000867

[23] Bell AW Bauman DE. Adaptations of glucose metabolism during pregnancy and lactation. J Mammary Gland Biol Neoplasia (1997) 2:265–78. 10.1023/A:1026336505343 10882310

[24] Lawrence R Lawrence R Breastfeeding Guide for the Medical Profession, Sixth Edition. Philadelphia, PA: Elsevier Mosby (2005).

[25] Augustine RA Ladyman SR Grattan DR. From feeding one to feeding many: hormone-induced changes in bodyweight homeostasis during pregnancy. J Physiol (2008) 586:387–97. 10.1113/jphysiol.2007.146316 18033810 PMC2375600

[26] Speakman JR. The cost of living: field metabolic rates of small mammals. Adv Ecol Res (2000) 30:177–297. 10.1242/jeb.099739 24920834

[27] Johnson MS Thomson SC Speakman JR. Limits to sustained energy intake. I. Lactation in the laboratory mouse *Mus musculus*. J Exp Biol (2001) 204:1925–35. 11441034 10.1242/jeb.204.11.1925

[28] Nguyen DA Beeman NE Neville MC Regulation of tight junction permeability in the mammary gland. In: Cereijido M Anderson J, editors. Tight Junctions. Boca Raton: CRC Press (2001). p. 395–414.

[29] Grattan DR Steyn FJ Kokay IC Anderson GM Bunn S. Pregnancy-induced adaptation in the neuroendocrine control of prolactin secretion. J Endocrinol (2008) 20:497–507. 10.1111/j.1365-2826.2008.01661.x 18266946

[30] Vernon RG Endocrine control of metabolic adaptation during lactation. Proc Nutr Soc (1989) 48:23–32 10.1079/PNS19890006 2660154

[31] Hapon MB Simoncini M Via G Jahn GA. Effect of hypothyroidism on hormone profiles in virgin, pregnant and lactating rats, and on lactation. Reproduction (2003) 126:371–82. 10.1530/rep.0.1260371 12968945

[32] Peters F Schulze-Tollert J Schuth W. Thyrotropin-releasing hormone – a lactation-promoting agent? Br J Obstet Gynaecol (1991) 98:880–5. 10.1111/j.1471-0528.1991.tb13509.x 1911606

[33] Bailey M Silver R. Sex differences in circadian timing systems: implications for disease. Front Neuroendocrinol (2014) 35:111–39. 10.1016/j.yfrne.2013.11.003 24287074 PMC4041593

[34] Bolon B Gender agenda: sex bias can be justified in animal research. Nature (2010) 466:28 10.1038/466028d 20595991

[35] Putting gender on the agenda. Nature (2010) 465:665 10.1038/465665a 20535156

[36] Baylis F Pregnant women deserve better. Nature (2010) 465:689–90 10.1038/465689a 20535185

[37] Plaut K Casey T. Does the circadian system regulate lactation? Animal (2012) 6:394–402. 10.1017/S1751731111002187 22436218

[38] Casey TM Plaut K. Lactation biology symposium: circadian clocks as mediators of the homeorhetic response to lactation. J Anim Sci (2012) 90:744–54. 10.2527/jas.2011-4590 22345106

[39] Reppert SM Weaver DR. Coordination of circadian timing in mammals. Nature (2002) 418:935–41. 10.1038/nature00965 12198538

[40] Moore RY Speh JC Serotonin innervation of the primate suprachiasmatic nucleus. Brain Res (2004) 1010:169–73 10.1016/j.brainres.2004.02.024 15126131

[41] Wirz-Justice A. Biological rhythm disturbances in mood disorders. Int Clin Psychopharmacol (2006) 21(Suppl 1):S11–5. 10.1097/01.yic.0000195660.37267.cf 16436934

[42] Sheward WJ Maywood ES French KL Horn JM Hastings MH Seckl JR Entrainment to feeding but not to light: circadian phenotype of VPAC2 receptor-null mice. J Neurosci (2007) 27:4351–8. 10.1523/JNEUROSCI.4843-06.2007 17442819 PMC6672325

[43] Hardin PE. Transcription regulation within the circadian clock: the E-box and beyond. J Biol Rhythms (2004) 19:348–60. 10.1177/0748730404268052 15534316

[44] Akhtar RA Reddy AB Maywood ES Clayton JD King VM Smith AG Circadian cycling of the mouse liver transcriptome, as revealed by cDNA microarray, is driven by the suprachiasmatic nucleus. Curr Biol (2002) 12:540–50. 10.1016/S0960-9822(02)00759-5 11937022

[45] Panda S. Coordinated transcription of key pathways in the mouse by the circadian clock. Cell (2002) 109:307–20. 10.1016/S0092-8674(02)00722-5 12015981

[46] Casey T Crodian J Donkin SS Plaut K Continuously changing light-dark phase decreases milk yield, fat, protein and lactose in dairy cows. J Adv Dairy Res (2014) 2:119 10.4172/2329-888X.1000119

[47] Gonnissen HK Hulshof T Westerterp-Plantenga MS. Chronobiology, endocrinology, and energy- and food-reward homeostasis. Obes Rev (2013) 14:405–16. 10.1111/obr.12019 23387351

[48] la Fleur SE Kalsbeek A Wortel J Fekkes ML Buijs RM. A daily rhythm in glucose tolerance: a role for the suprachiasmatic nucleus. Diabetes (2001) 50:1237–43. 10.2337/diabetes.50.6.1237 11375322

[49] Reiter RJ Tan DX Korkmaz A Erren TC Piekarski C Tamura H Light at night, chronodisruption, melatonin suppression, and cancer risk: a review. Crit Rev Oncog (2007) 13:303–28. 10.1615/CritRevOncog.v13.i4.30 18540832

[50] Pauley SM. Lighting for the human circadian clock: recent research indicates that lighting has become a public health issue. Med Hypotheses (2004) 63:588–96. 10.1016/j.mehy.2004.03.020 15325001

[51] Díaz-Muñoz M Vázquez-Martínez O Aguilar-Roblero R Escobar C. Anticipatory changes in liver metabolism and entrainment of insulin, glucagon, and corticosterone in food-restricted rats. Am J Physiol (2000) 279:R2048–56. 11080068 10.1152/ajpregu.2000.279.6.R2048

[52] Milano W De Rosa M Milano L Capasso A Night eating syndrome: an overview. J Pharm Pharmacol (2012) 64:2–10 10.1111/j.2042-7158.2011.01353.x 22150667

[53] Goel N Stunkard AJ Rogers NL Van Dongen HPA Allison KC O’Reardon JP Circadian rhythm profiles in women with night eating syndrome. J Biol Rhythms (2009) 24:85–94. 10.1177/0748730408328914 19150931 PMC3564642

[54] Bass J Takahashi JS. Circadian integration of metabolism and energetics. Science (2010) 330:1349–54. 10.1126/science.1195027 21127246 PMC3756146

[55] Gimble JM Bray MS Young A. Circadian biology and sleep: missing links in obesity and metabolism? Obes Rev (2009) 10:1–5. 10.1111/j.1467-789X.2009.00672.x 19849796

[56] Karlsson B Knutsson A Lindahl B. Is there an association between shift work and having a metabolic syndrome? Results from a population based study of 27,485 people. Occup Environ Med (2001) 58:747–52. 10.1136/oem.58.11.747 11600731 PMC1740071

[57] Lamia KA Storch KF Weitz CJ. Physiological significance of a peripheral tissue circadian clock. Proc Natl Acad Sci U S A (2008) 105:15172–7. 10.1073/pnas.0806717105 18779586 PMC2532700

[58] Suwazono Y Dochi M Sakata K Okubo Y Oishi M Tanaka K A longitudinal study on the effect of shift work on weight gain in male Japanese workers. Obesity (Silver Spring) (2008) 16:1887–93. 10.1038/oby.2008.298 18535539

[59] Boivin DB Boudreau P. Impacts of shift work on sleep and circadian rhythms. Pathol Biol (Paris) (2014) 62:292–301. 10.1016/j.patbio.2014.08.001 25246026

[60] Froy O. The relationship between nutrition and circadian rhythms in mammals. Front Neuroendocrinol (2007) 28:61–71. 10.1016/j.yfrne.2007.03.001 17451793

[61] Foster RG Wulff K. The rhythm of rest and excess. Nat Rev Neurosci (2005) 6:407–14. 10.1038/nrn1670 15861183

[62] Scheer FAJL Hilton MF Mantzoros CS Shea SA. Adverse metabolic and cardiovascular consequences of circadian misalignment. Proc Natl Acad Sci U S A (2009) 106:4453–8. 10.1073/pnas.0808180106 19255424 PMC2657421

[63] Sookoian S Castano G Gemma C Gianotti TF Pirola CJ. Common genetic variations in CLOCK transcription factor are associated with nonalcoholic fatty liver disease. World J Gastroenterol (2007) 13:4242–8. 17696255 10.3748/wjg.v13.i31.4242 PMC4250625

[64] Woon PY Kaisaki PJ Braganca J Bihoreau MT Levy JC Farrall M Aryl hydrocarbon receptor nuclear translocator-like (BMAL1) is associated with susceptibility to hypertension and type 2 diabetes. Proc Natl Acad Sci U S A (2007) 104:14412–7. 10.1073/pnas.0703247104 17728404 PMC1958818

[65] Ando H Yanagihara H Hayashi Y Obi Y Tsuruoka S Takamura T Rhythmic messenger ribonucleic acid expression of clock genes and adipocytokines in mouse visceral adipose tissue. Endocrinology (2005) 146:5631–6. 10.1210/en.2005-0771 16166217

[66] Mendoza J Pévet P Challet E. High-fat feeding alters the clock synchronization to light. J Physiol (2008) 586:5901–10. 10.1113/jphysiol.2008.159566 18936083 PMC2655413

[67] Martin RJ Wangsness PJ Gahagan JH. Diurnal changes in serum metabolites and hormones in lean and obese Zucker rats. Horm Metab Res (1978) 10:187–92. 10.1055/s-0028-1093431 566707

[68] Pasquali R Vicennati V Cacciari M Pagotto U. The hypothalamic-pituitary-adrenal axis activity in obesity and the metabolic syndrome. Ann N Y Acad Sci (2006) 1083:111–28. 10.1196/annals.1367.009 17148736

[69] Dardente H Wyse CA Birnie MJ Dupré SM Loudon ASI Lincoln GA A molecular switch for photoperiod responsiveness in mammals. Curr Biol (2010) 20:2193–8. 10.1016/j.cub.2010.10.048 21129971

[70] Navara KJ Nelson RJ. The dark side of light at night: physiological, epidemiological, and ecological consequences. J Pineal Res (2007) 43:215–24. 10.1111/j.1600-079X.2007.00473.x 17803517

[71] Obayashi K Saeki K Iwamoto J Okamoto N Tomioka K Nezu S Exposure to light at night, nocturnal urinary melatonin excretion, and obesity/dyslipidemia in the elderly: a cross-sectional analysis of the HEIJO-KYO study. J Clin Endocrinol Metab (2013) 98:337–44. 10.1210/jc.2012-2874 23118419

[72] McFadden E Jones ME Schoemaker MJ Ashworth A Swerdlow AJ. The relationship between obesity and exposure to light at night: cross-sectional analyses of over 100,000 women in the breakthrough generations study. Am J Epidemiol (2014) 180:245–50. 10.1093/aje/kwu117 24875371

[73] Medicine IO Sleep Disorders and Sleep Deprivation: An Unmet Public Health Problem. Washington, DC: The National Academies Press (2006). 20669438

[74] Schoenborn CA Adams PF Health behaviors of adults: United States, 2005–2007 national center for health statistics. Vital Health Stat (2010) 10(245). Available from: http://www.cdc.gov/nchs/data/series/sr_10/sr10_245.pdf 20669609

[75] Spiegel KLR Van Cauter E. Impact of sleep debt on metabolic and endocrine function. Lancet (1999) 354:1435–9. 10.1016/S0140-6736(99)01376-8 10543671

[76] Breslau N Roth T Rosenthal L Andreski P. Sleep disturbance and psychiatric disorders: a longitudinal epidemiological study of young adults. Biol Psychiatry (1996) 39:411–8. 10.1016/0006-3223(95)00188-3 8679786

[77] Bjorvatn B Sagen IM Oyane N Waage S Fetveit A Pallesen S The association between sleep duration, body mass index and metabolic measures in the Hordaland Health Study. J Sleep Res (2007) 16:66–76. 10.1111/j.1365-2869.2007.00569.x 17309765

[78] Heslop P Smith GD Metcalfe C Macleod J Hart C. Sleep duration and mortality: the effect of short or long sleep duration on cardiovascular and all-cause mortality in working men and women. Sleep Med (2002) 3:305–14. 10.1016/S1389-9457(02)00016-3 14592192

[79] Cappuccio FP Stranges S Kandala NB Miller MA Taggart FM Kumari M Gender-specific associations of short sleep duration with prevalent and incident hypertension: the Whitehall II Study. Hypertension (2007) 50:693–700. 10.1161/HYPERTENSIONAHA.107.095471 17785629 PMC3221967

[80] Meisinger C Heier M Lowel H Schneider A Doring A. Sleep duration and sleep complaints and risk of myocardial infarction in middle-aged men and women from the general population: the MONICA/KORA Augsburg cohort study. Sleep (2007) 30:1121–7. 17910384 10.1093/sleep/30.9.1121 PMC1978404

[81] Broussard J Brady MJ. The impact of sleep disturbances on adipocyte function and lipid metabolism. Best Pract Res Clin Endocrinol Metab (2010) 24:763–73. 10.1016/j.beem.2010.08.007 21112024 PMC3031100

[82] Buxton OM Cain SW O’Connor SP Porter JH Duffy JF Wang W Adverse metabolic consequences in humans of prolonged sleep restriction combined with circadian disruption. Sci Transl Med (2012) 4:129ra143. 10.1126/scitranslmed.3003200 22496545 PMC3678519

[83] Bailey SM Udoh US Young ME. Circadian regulation of metabolism. J Endocrinol (2014) 222:R75–96. 10.1530/JOE-14-0200 24928941 PMC4109003

[84] Karatsoreos IN Bhagat S Bloss EB Morrison JH McEwen BS. Disruption of circadian clocks has ramifications for metabolism, brain, and behavior. Proc Natl Acad Sci U S A (2011) 108:1657–62. 10.1073/pnas.1018375108 21220317 PMC3029753

[85] Reutrakul S Van Cauter E. Interactions between sleep, circadian function, and glucose metabolism: implications for risk and severity of diabetes. Ann N Y Acad Sci (2014) 1311:151–73. 10.1111/nyas.12355 24628249

[86] Husse J Hintze SC Eichele G Lehnert H Oster H. Circadian clock genes Per1 and Per2 regulate the response of metabolism-associated transcripts to sleep disruption. PLoS One (2012) 7:e52983. 10.1371/journal.pone.0052983 23285241 PMC3532432

[87] Morris CJ Aeschbach D Scheer FA Circadian system, sleep and endocrinology. Mol Cell Endocrinol (2012) 349:91–104 10.1016/j.mce.2011.09.003 21939733 PMC3242827

[88] Zee PC Attarian H Videnovic A Circadian rhythm abnormalities. Continuum (Minneap Minn) (2013) 19:132–47 10.1212/01.CON.0000427209.21177.aa 23385698 PMC3654533

[89] Gangwisch JE. Epidemiological evidence for the links between sleep, circadian rhythms and metabolism. Obes Rev (2009) 10:37–45. 10.1111/j.1467-789X.2009.00663.x 19849800 PMC4075056

[90] Miller BH Takahashi JS Central circadian control of female reproductive function. Front Endocrinol (2014) 4:195 10.3389/fendo.2013.00195 PMC3898595 24478756

[91] Olcese J Circadian clocks and pregnancy. Front Endocrinol (2014) 5:123 10.3389/fendo.2014.00123 PMC4109505 25104949

[92] Sellix MT. Clocks underneath: the role of peripheral clocks in the timing of female reproductive physiology. Front Endocrinol (2013) 4:91. 10.3389/fendo.2013.00091 23888155 PMC3719037

[93] Kriegsfeld LJ Williams I Wilbur P. Circadian control of the estrogenic circuits regulating GnRH secretion and the preovulatory luteinizing hormone surge. Front Endocrinol (Lausanne) (2012) 3:60. 10.3389/fendo.2012.00060 22661968 PMC3356853

[94] Turek FW Swann J Earnest DJ Role of the circadian system in reproductive phenomena. Recent Prog Horm Res (1984) 40:143–83. 6148772 10.1016/b978-0-12-571140-1.50009-8

[95] Dolatshad H Campbell EA O’Hara L Maywood ES Hastings MH Johnson MH. Developmental and reproductive performance in circadian mutant mice. Hum Reprod (2006) 21:68–79. 10.1093/humrep/dei313 16210390

[96] Pilorz V Steinlechner S. Low reproductive success in Per1 and Per2 mutant mouse females due to accelerated ageing? Reproduction (2008) 135:559–68. 10.1530/REP-07-0434 18367514

[97] Sellix M Menaker M. Circadian clocks in mammalian reproductive physiology: effects of the “other” biological clock on fertility. Discov Med (2011) 11:273–81. 21524381

[98] Leclerc GM Boockfor FR. Pulses of prolactin promoter activity depend on a noncanonical E-box that can bind the circadian proteins CLOCK and BMAL1. Endocrinology (2005) 146:2782–90. 10.1210/en.2005-0100 15774559

[99] Baumgartner A Dietzel M Saletu B Wolf R Campos-Barros A Graf KJ Influence of partial sleep deprivation on the secretion of thyrotropin, thyroid hormones, growth hormone, prolactin, luteinizing hormone, follicle stimulating hormone, and estradiol in healthy young women. Psychiatry Res (1993) 48:153–78. 10.1016/0165-1781(93)90039-J 8416024

[100] Kittrell EM Satinoff E. Diurnal rhythms of body temperature, drinking and activity over reproductive cycles. Physiol Behav (1988) 42:477–84. 10.1016/0031-9384(88)90180-1 3393610

[101] Scribner SJ Wynne-Edwards KE. Disruption of body temperature and behavior rhythms during reproduction in dwarf hamsters (*Phodopus*). Physiol Behav (1994) 55:361–9. 10.1016/0031-9384(94)90147-3 8153179

[102] Schrader JA Nunez AA Smale L. Changes in and dorsal to the rat suprachiasmatic nucleus during early pregnancy. Neuroscience (2010) 171:513–23. 10.1016/j.neuroscience.2010.08.057 20807562 PMC2975742

[103] Schrader JA Smale L Nunez AA. Pregnancy affects FOS rhythms in brain regions regulating sleep/wake state and body temperature in rats. Brain Res (2012) 1480:53–60. 10.1016/j.brainres.2012.09.003 22975436 PMC4124522

[104] Schrader JA Walaszczyk EJ Smale L. Changing patterns of daily rhythmicity across reproductive states in diurnal female Nile grass rats (*Arvicanthis niloticus*). Physiol Behav (2009) 98:547–56. 10.1016/j.physbeh.2009.08.012 19744504 PMC2783347

[105] Kimura M Zhang SQ Inoue S Pregnancy-associated sleep changes in the rat. Am J Physiol Regul Integr Comp Physiol (1996) 271:R1063–9. 10.1152/ajpregu.1996.271.4.R1063 8898001

[106] Suzuki S Dennerstein L Greenwood KM Armstrong SM Satohisa E. Sleeping patterns during pregnancy in Japanese women. J Psychosom Obstet Gynaecol (1994) 15:19–26. 10.3109/01674829409025625 8038885

[107] Facco FL Kramer J Ho KH Zee PC Grobman WA Sleep disturbances in pregnancy. Obstet Gynecol (2010) 115:77–83 10.1097/AOG.0b013e3181c4f8ec 20027038

[108] Okun ML Coussons-Read ME. Sleep disruption during pregnancy: how does it influence serum cytokines? J Reprod Immunol (2007) 73:158–65. 10.1016/j.jri.2006.06.006 17074396

[109] Posadas ES Meliska CJ Martinez LF Sorenson DL Lopez AM Nowakowski S The relationship of nocturnal melatonin to estradiol and progesterone in depressed and healthy pregnant women. J Womens Health (Larchmt) (2012) 21:649–55. 10.1089/jwh.2011.3191 22320439 PMC3366092

[110] Parry BL Meliska CJ Sorenson DL Lopez AM Martinez LF Nowakowski S Plasma melatonin circadian rhythm disturbances during pregnancy and postpartum in depressed women and women with personal or family histories of depression. Am J Psychiatry (2008) 165:1551–8. 10.1176/appi.ajp.2008.08050709 18829869 PMC3038788

[111] Allolio B Hoffmann J Linton EA Winkelmann W Kusche M. Diurnal salivary cortisol patterns during pregnancy and after delivery: relationship to plasma corticotrophin-releasing-hormone. Clin Endocrinol (Oxf) (1990) 33:279–89. 10.1111/j.1365-2265.1990.tb00492.x 2225483

[112] Goodman RL Jansen HT Billings HJ Coolen LM Lehman MN. Neural systems mediating seasonal breeding in the Ewe. J Neuroendocrinol (2010) 22:674–81. 10.1111/j.1365-2826.2010.02014.x 20456601 PMC2908208

[113] Entringer S Buss C Shirtcliff EA Cammack AL Yim IS Chicz-DeMet A Attenuation of maternal psychophysiological stress responses and the maternal cortisol awakening response over the course of human pregnancy. Stress (2010) 13:258–68. 10.3109/10253890903349501 20067400 PMC2862645

[114] Lightman SL Windle RJ Wood SA Kershaw YM Shanks N Ingram CD. Peripartum plasticity within the hypothalamo-pituitary-adrenal axis. Prog Brain Res (2001) 133:111–29. 10.1016/S0079-6123(01)33009-1 11589125

[115] Tu M Lupien S Walker C. Diurnal salivary cortisol levels in postpartum mothers as a function of infant feeding choice and parity. Psychoneuroendocrinology (2006) 31:812–24. 10.1016/j.psyneuen.2006.03.006 16716531

[116] Reddy AB Maywood ES Karp NA King VM Inoue Y Gonzalez FJ Glucocorticoid signaling synchronizes the liver circadian transcriptome. Hepatology (2007) 45:1478–88. 10.1002/hep.21571 17538967

[117] Cooperstock M England JE Wolfe RA. Circadian incidence of labor onset hour in preterm birth and chorioamnionitis. Obstet Gynecol (1987) 70:852–5. 3684119

[118] Cooperstock M England JE Wolfe RA. Circadian incidence of premature rupture of the membranes in term and preterm births. Obstet Gynecol (1987) 69:936–41. 3574825

[119] Ngwenya S Lindow SW. 24 hour rhythm in the timing of pre-labour spontaneous rupture of membranes at term. Eur J Obstet Gynecol Reprod Biol (2004) 112:151–3. 10.1016/S0301-2115(03)00286-0 14746949

[120] Mancuso PJ Alexander JM McIntire DD Davis E Burke G Leveno KJ. Timing of birth after spontaneous onset of labor. Obstet Gynecol (2004) 103:653–6. 10.1097/01.AOG.0000118309.70035.63 15051554

[121] Dodd JM Crowther CA Robinson JS. Morning compared with evening induction of labor: a nested randomized controlled trial. A nested randomized controlled trial. Obstet Gynecol (2006) 108:350–60. 10.1097/01.AOG.0000227746.35565.d9 16880306

[122] Freeman ME Kanyicska B Lerant A Nagy G. Prolactin: structure, function, and regulation of secretion. Physiol Rev (2000) 80:1523–631. 11015620 10.1152/physrev.2000.80.4.1523

[123] Casey TM Crodian J Erickson E Kuropatwinski KK Gleiberman AS Antoch MP. Tissue-specific changes in molecular clocks during the transition from pregnancy to lactation in mice. Biol Reprod (2014) 90(6):127. 10.1095/biolreprod.113.116137 24759789 PMC4094001

[124] Schradera J Nunez A Smale L. Changes in and dorsal to the rat suprachiasmatic nucleus during early pregnancy. Neuroscience (2010) 171:513–23. 10.1016/j.neuroscience.2010.08.057 20807562 PMC2975742

[125] Maningat PD Sen P Rijnkels M Sunehag AL Hadsell DL Bray M Gene expression in the human mammary epithelium during lactation: the milk fat globule transcriptome. Physiol Genomics (2009) 37:12–22. 10.1152/physiolgenomics.90341.2008 19018045 PMC2661101

[126] Miller BH Olson SL Turek FW Levine JE Horton TH Takahashi JS. Circadian clock mutation disrupts estrous cyclicity and maintenance of pregnancy. Curr Biol (2004) 14:1367–73. 10.1016/j.cub.2004.07.055 15296754 PMC3756147

[127] Dahl GE. Effects of short day photoperiod on prolactin signaling in dry cows: a common mechanism among tissues and environments? J Anim Sci (2008) 86:10–4. 10.2527/jas.2007-0311 17686892

[128] Summa KC Vitaterna MH Turek FW. Environmental perturbation of the circadian clock disrupts pregnancy in the mouse. PLoS One (2012) 7:e37668. 10.1371/journal.pone.0037668 22649550 PMC3359308

[129] Varcoe TJ Boden MJ Voultsios A Salkeld MD Rattanatray L Kennaway DJ. Characterisation of the maternal response to chronic phase shifts during gestation in the rat: implications for fetal metabolic programming. PLoS One (2013) 8:e53800. 10.1371/journal.pone.0053800 23342007 PMC3544759

[130] Varcoe T Wight N Voultsios A Salkeld M Kennaway D. Chronic phase shifts of the photoperiod throughout pregnancy programs glucose intolerance and insulin resistance in the rat. PLoS One (2011) 6:e18504. 10.1371/journal.pone.0018504 21494686 PMC3071829

[131] Kovanen L Saarikoski ST Aromaa A Lönnqvist J Partonen T. *ARNTL* (*BMAL1*) and *NPAS2* gene variants contribute to fertility and seasonality. PLoS One (2010) 5:e10007. 10.1371/journal.pone.0010007 20368993 PMC2848852

[132] Knutsson A Health disorders of shift workers. Occup Med (2003) 53:103–8 10.1093/occmed/kqg048 12637594

[133] Zhu JL Hjollund NH Andersen AM Olsen J. Shift work, job stress, and late fetal loss: the national birth cohort in Denmark. J Occup Environ Med (2004) 46:1144–9. 10.1097/01.jom.0000145168.21614.21 15534501

[134] Zhu JL Hjollund NH Olsen J. Shift work, duration of pregnancy, and birth weight: the national birth cohort in Denmark. Am J Obstet Gynecol (2004) 191:285–91. 10.1016/j.ajog.2003.12.002 15295380

[135] Abeysena C Jayawardana P de A Seneviratne R. Maternal sleep deprivation is a risk factor for small for gestational age: a cohort study. Aust N Z J Obstet Gynaecol (2009) 49:382–7. 10.1111/j.1479-828X.2009.01010.x 19694692

[136] Williams MA Miller RS Qiu C Cripe SM Gelaye B Enquobahrie D. Associations of early pregnancy sleep duration with trimester-specific blood pressures and hypertensive disorders in pregnancy. Sleep (2010) 33:1363–71. 21061859 10.1093/sleep/33.10.1363 PMC2941423

[137] Izci Balserak B Jackson N Ratcliffe S Pack A Pien G. Sleep-disordered breathing and daytime napping are associated with maternal hyperglycemia. Sleep Breath (2013) 17(3):1093–102. 10.1007/s11325-013-0809-4 23354511 PMC3696035

[138] Qiu C Enquobahrie D Frederick I Abetew D Williams M. Glucose intolerance and gestational diabetes risk in relation to sleep duration and snoring during pregnancy: a pilot study. BMC Womens Health (2010) 10:17. 10.1186/1472-6874-10-17 20470416 PMC2885310

[139] Mendieta Zeron H Garcia Solorio VJ Nava Diaz PM Garduno Alanis A Santillan Benitez JG Dominguez Garcia V Hyperleptinemia as a prognostic factor for preeclampsia: a cohort study. Acta Medica (Hradec Kralove) (2012) 55:165–71. 23631287 10.14712/18059694.2015.41

[140] Henson MC Castracane VD. Leptin in pregnancy: an update. Biol Reprod (2006) 74:218–29. 10.1095/biolreprod.105.045120 16267210

[141] Kocyigit Y Bayhan G Atamer A Atamer Y. Serum levels of leptin, insulin-like growth factor-I and insulin-like growth factor binding protein-3 in women with pre-eclampsia, and their relationship to insulin resistance. Gynecol Endocrinol (2004) 18:341–8. 10.1080/09513590410001704975 15497497

[142] Qiu C Frederick IO Sorensen TK Enquobahrie DA Williams MA. Sleep duration and plasma leptin concentrations in early pregnancy among lean and overweight/obese women: a cross sectional study. BMC Res Notes (2014) 7:20. 10.1186/1756-0500-7-20 24405869 PMC3896691

[143] McClung C. Circadian genes, rhythms and the biology of mood disorders. Pharmacol Ther (2007) 114:222–32. 10.1016/j.pharmthera.2007.02.003 17395264 PMC1925042

[144] Monteleone P Maj M. The circadian basis of mood disorders: recent developments and treatment implications. Eur Neuropsychopharmacol (2008) 18:701–11. 10.1016/j.euroneuro.2008.06.007 18662865

[145] Dennis CL McQueen K. Does maternal postpartum depressive symptomatology influence infant feeding outcomes? Acta Paediatr (2007) 96:590–4. 10.1111/j.1651-2227.2007.00184.x 17391475

[146] Shaw MA Rasmussen KM Myers TR. Consumption of a high fat diet impairs reproductive performance in Sprague-Dawley rats. J Nutr (1997) 127(1):64–9. 9040546 10.1093/jn/127.1.64

[147] Flint DJ Travers MT Barber MC Binart N Kelly PA. Diet-induced obesity impairs mammary development and lactogenesis in murine mammary gland. Am J Physiol Endocrinol Metab (2005) 288:E1179–87. 10.1152/ajpendo.00433.2004 15671082

[148] Agius L Rolls BJ Rowe EA Williamson DH. Obese rats develop hyperketonemia and a fatty liver during lactation. Int J Obes (1983) 7:447–52. 6358070

[149] Reddy TE Gertz J Crawford GE Garabedian MJ Myers RM. The hypersensitive glucocorticoid response specifically regulates period 1 and expression of circadian genes. Mol Cell Biol (2012) 32:3756–67. 10.1128/MCB.00062-12 22801371 PMC3430195

[150] Casey T Plaut K. The role of glucocorticoids in secretory activation and milk secretion, a historical perspective. J Mammary Gland Biol Neoplasia (2007) 12:293–304. 10.1007/s10911-007-9055-3 18000742

[151] Henderson JJ Hartmann PE Moss TJM Doherty DA Newnham JP. Disrupted secretory activation of the mammary gland after antenatal glucocorticoid treatment in sheep. Reproduction (2008) 136:649–55. 10.1530/REP-08-0134 18663017

[152] Amaral FG Castrucci AM Cipolla-Neto J Poletini MO Mendez N Richter HG Environmental control of biological rhythms: effects on development, fertility and metabolism. J Neuroendocrinol (2014) 26:603–12. 10.1111/jne.12144 24617798

[153] Meedya S Fahy K Kable A. Factors that positively influence breastfeeding duration to 6 months: a literature review. Women Birth (2010) 23(4):135–45. 10.1016/j.wombi.2010.02.002 20299299

[154] Fairbank L O’Meara S Renfrew MJ Woolridge M Sowden AJ Lister-Sharp D. A systematic review to evaluate the effectiveness of interventions to promote the initiation of breastfeeding. Health Technol Assess (2000) 4:1–171. 10.3310/hta4250 11111103

[155] Shealy KR Li R Benton-Davis S Grummer-Strawn LM The CDC Guide to Breastfeeding Interventions. U.S. Department of Health and Human Services, Centers for Disease Control and Prevention (2005). Available from: http://www.cdc.gov/breastfeeding/pdf/breastfeeding_interventions.pdf

[156] Leproult R Deliens G Gilson M Peigneux P. Beneficial impact of sleep extension on fasting insulin sensitivity in adults with habitual sleep restriction. Sleep (2014). 25348128 10.5665/sleep.4660 PMC4402666

[157] The Baby Friendly Hospital Initiative. Guidelines and Evaluation Criteria for Facilities Seeking Baby-Friendly Designation. (2010). Available from: www.babyfriendlyusa.org/

[158] Blackburn S Environmental impact of the NICU on developmental outcomes. J Pediatr Nurs (1998) 13:279–89 10.1016/S0882-5963(98)80013-4 9798363

[159] Mann NP Haddow R Stokes L Goodley S Rutter N. Effect of night and day on preterm infants in a newborn nursery: randomised trial. Br Med J (Clin Res Ed) (1986) 293:1265–7. 10.1136/bmj.293.6557.1265 3096460 PMC1342106

[160] Vásquez-Ruiz S Maya-Barrios JA Torres-Narváez P Vega-Martínez BR Rojas-Granados A Escobar C A light/dark cycle in the NICU accelerates body weight gain and shortens time to discharge in preterm infants. Early Hum Dev (2014) 90:535–40. 10.1016/j.earlhumdev.2014.04.015 24831970

[161] Casper RF Rahman S. Spectral modulation of light wavelengths using optical filters: effect on melatonin secretion. Fertil Steril (2014) 102:336–8. 10.1016/j.fertnstert.2014.06.006 25015557

